# Patterns of acquired HIV-1 drug resistance mutations and predictors of virological failure in Moshi, Northern Tanzania

**DOI:** 10.1371/journal.pone.0232649

**Published:** 2020-09-28

**Authors:** Shabani Ramadhani Mziray, Happiness H. Kumburu, Hellen B. Assey, Tolbert B. Sonda, Michael J. Mahande, Sia E. Msuya, Ireen E. Kiwelu

**Affiliations:** 1 Department of Biochemistry and Molecular Biology, Kilimanjaro Christian Medical University College (KCMUCo), Moshi, Kilimanjaro, Tanzania; 2 Department of Medical Laboratory Services, Kibong’oto Infectious Diseases Hospital (KIDH), Siha, Kilimanjaro, Tanzania; 3 Kilimanjaro Clinical Research Institute (KCRI), Moshi, Kilimanjaro, Tanzania; 4 Department of Epidemiology and Biostatistics, Institute of Public Health, Kilimanjaro Christian Medical University College (KCMUCo), Moshi, Kilimanjaro, Tanzania; 5 Department of Community Health, Institute of Public Health, Kilimanjaro Christian Medical University College (KCMUCo), Moshi, Kilimanjaro, Tanzania; University of Cincinnati College of Medicine, UNITED STATES

## Abstract

Emergence of HIV drug resistance poses a serious risk of inactivity to all currently approved antiretroviral drugs. Profiles of HIV drug resistance mutations (HIVDRM) and virological failure (VF) are not extensively studied in Tanzania. This study aimed to determine HIVDRM and predictors of VF in HIV-infected individuals failing first-line HIV drugs in Moshi, Northern Tanzania. A case-control study was conducted at Kilimanjaro Christian Medical Centre, Mawenzi, Pasua and Majengo health facilities with HIV-care and treatment clinics from October, 2017 to August, 2018. Cases and controls were HIV-infected individuals with VF and viral suppression (VS) respectively. HIV-1 reverse transcriptase and protease genes were amplified and sequenced. Stanford University’s HIV drug resistance database and REGA subtyping tool 3.0 determined HIVDRM and HIV-1 subtypes respectively. Odds ratios (OR) with 95% confidence interval (95% CI) investigated predictors of VF. *P*-value < 5% was considered statistically significant. A total of 124 participants were recruited, of whom 63 (50.8%) had VF, 61 (49.2%) had VS and 82 (66.1%) were females. Median [IQR] age and duration on ART were 45 [35–52] years and 72 [48–104] months respectively. Twenty-five out of 26 selected samples from cases were successfully sequenced. Twenty-four samples (96%) had at least one major mutation conferring resistance to HIV drugs, with non-nucleoside analogue reverse transcriptase inhibitor (NNRTI)-resistance associated mutations as the majority (92%). Frequent NNRTI-resistance associated mutations were K103N (n = 11), V106M (n = 5) and G190A (n = 5). Prevalent nucleoside analogue reverse transcriptase inhibitors-resistance associated mutations were M184V (n = 17), K70R (n = 7) and D67N (n = 6). Dual-class resistance was observed in 16 (64%) samples. Thirteen samples (52%) had at least one thymidine analogue-resistance associated mutation (TAM). Three samples (12%) had T69D mutation with at least 1 TAM. Two samples (8%) had at least one mutation associated with protease inhibitor resistance. Age [aOR = 0.94, 95% CI (0.90–0.97), *p* < 0.001] and occupation [aOR = 0.35, 95% CI (0.12–1.04), *p* = 0.059] associated with VF. In conclusion, HIV drug resistance is common among people failing antiretroviral therapy. Resistance testing will help to guide switching of HIV drugs.

## Introduction

Human Immunodeficiency Virus (HIV) infection is a significant public health problem with an estimation of 37.9 million people living with HIV worldwide by the end of 2018. Among the people living with HIV (PLHIV) globally, 36.2 million are adults, 1.7 million are children below 15 years and 18.8 million are women aged 15 years and above. Every day, 5000 people are infected with HIV globally, with more than half of these new infections in sub-Saharan Africa (sSA). More than half of the global burden of HIV-infections are in Eastern and Southern Africa with 20.6 million PLHIV by the year 2018 [1]. The incidence-prevalence ratio (IPR) of HIV globally has declined considerably from 11.2% in 2000 to 4.6% in 2018. In the same year, HIV IPR in sSA declined to about 3.9% and 5.5% in Eastern and Southern Africa; and Western and Central Africa respectively. Despite the decline in IPR, HIV-infection remains a public health problem in sSA with approximately 68% of the global burden of PLHIV and a substantial number of new HIV-infections [1].

Highly active antiretroviral therapy (HAART) in sSA countries including Tanzania has helped to reduce HIV/AIDS-related mortality from around 900,000 in 2010 to 470,000 in 2018 [1] with improved quality of life [2]. The “90-90-90 target”, set by the United Nations Member States, aims for 90% of PLHIV to know their HIV status, 90% of PLHIV who know their HIV status to be on ART, and also 90% of PLHIV on ART to have sustainable viral suppression by the year 2020 [3]. In the race to achieve the second UNAIDS ‘90’ target, about 51% of PLHIV are on HAART in Western and Central Africa (WCA); and 67% in Eastern and Southern Africa (ESA). The proportions of HIV-infected individuals on treatment with viral suppression is better in WCA (79%) compared to ESA (58%); however, both proportions are below the third UNAIDS ‘90’ target [1]. One of the key barriers to achieve the third UNAIDS ‘90’ in sSA is the emergence of HIV drug resistance (HIVDR).

HIVDR is defined by the World Health Organisation (WHO) as the presence of one or more mutations in HIV drug-targeted genes that compromise the ability of a specific drug or combination of drugs to block replication of HIV [4]. HIV-infected individuals failing first-line HAART regimens are reported to have 50 to 97% of non-nucleoside analogue reverse transcriptase inhibitors (NNRTI) resistance worldwide [4]. In sSA, more than 80% of HIV-infected individuals with virological failure (VF) on first-line HAART have HIV-drug resistance [5]. Also, sSA has been reported to accommodate tenofovir resistance in more than a half of people with first-line HAART failure on tenofovir-based regimens. Cytosine analogue resistance is common in sSA as compared to Western Europe. Eastern Africa more commonly reported lamivudine and emtricitabine resistance than NNRTI resistance [6].

Tanzania introduced antiretroviral therapy (ART) program in 2004. As of 2016, 839,544 people were on ART in Tanzania. The recommended triple first-line ART was 2 nucleoside analogue reverse transcriptase inhibitors (NRTI) + 1 NNRTI or 2 NRTI + 1 PI. The preferred (default) first-line ART regimen for adults, adolescents, pregnant/lactating mothers at the time of the present study was tenofovir (TDF) + lamivudine (3TC) + efavirenz (EFV). Prescription of ART at initiation and when switching is based on several factors, including co-morbidities, clinical condition, co-administered drugs, pregnancy status, age, and drug tolerability. Other recommended first-line ART combination regimens at the time of the study included TDF + emtricitabine (FTC) + EFV; TDF + (3TC or FTC) + dolutegravir (DTG); abacavir (ABC) + 3TC + (EFV or DTG); zidovudine (AZT) + 3TC + (EFV or DTG); AZT + 3TC + nevirapine (NVP); TDF + (FTC or 3TC) + atazanavir boosted by ritonavir (ATV/r). In case of first-line HAART failure, the recommended second-line HAART for adults and adolescents was zidovudine/lamivudine + ritonavir-boosted atazanavir or tenofovir/emtricitabine + ritonavir-boosted atazanavir [7]. In Tanzania, the markers for treatment failure are based on immunological, clinical and WHO recommended virological criteria [8]. Clinical and immunological criteria were extensively described to be less sensitive and less effective [9] and may reduce early notifications of VF. Tanzania continues to scale-up HIV viral load (HVL) testing which is currently done at a few selected settings. Of more public health importance is that HIV-infected individuals confirmed to have VF are switched to second-line HAART without programmatic HIV drug resistance testing and monitoring, a practice which may transfer cross-resistance patterns to newly switched drugs and limit available treatment options.

A wide range of first-line HIV drug resistance mutations (HIVDRMs) has been reported in Tanzania, including the thymidine analogue associated mutations which compromise susceptibility to multi-NRTIs [10–12]. To complement the efforts of unravelling the burden of HIVDR and preserve the integrity of limited second-line HIV drugs in Tanzania, this study aimed to examine genotypic drug resistance in reverse transcriptase (RT) and protease genes from selected individuals with VF on a wide range of first-line HAART in Moshi municipality, Northern Tanzania. The study also explored independent predictions for VF.

## Materials and methods

### Study design and settings

This was an unmatched case-control study done in four HIV/AIDS care and treatment clinics (CTC) in Moshi municipality, situated in Kilimanjaro region, Northern Tanzania from October to August 2018. Moshi municipality is one of the seven districts of Kilimanjaro region. The municipality has a total of 18 health facilities with CTC. Four out of 18 CTCs with high client volume were purposively selected to participate in the study. CTCs included were Kilimanjaro Christian Medical Centre (KCMC), Mawenzi Regional Referral Hospital, Majengo and Pasua Health Centres. KCMC provides tertiary care medical services to around 6.8 million people living in the northern zone of Tanzania (Tanga, Kilimanjaro, Arusha and Manyara) and other referrals from nearby settings. Mawenzi Regional Referral Hospital provides referral medical services to approximately 1.6 million people in the Kilimanjaro region.

At the time of study recruitment, management of HIV-infected individuals was done in-line with National Guidelines for the management of HIV and AIDS of 2017 [8]. CTCs routinely provided HIV counselling and testing services, ART care and treatment, treatment monitoring, laboratory investigations and treatment adherence support to HIV-infected individuals. Prescription of antiretroviral drugs was done by clinicians and in some cases by trained nurses in the health centres. HVL testing was routinely done for all HIV-infected individuals who have been on ART for at least 6 months. Those found with HVL > 1,000 copies/ml were offered enhanced adherence counselling (EAC) by trained health care providers on a monthly basis for three months followed by retesting of HVL to confirm VF. Clinic visits for adolescents and youths were scheduled at different times from adults to maximize adherence counselling and support. Drug adherence levels of ≥ 95% and < 95% was regarded as good and poor respectively [8]. Drug adherence was monitored by pill counting during the participant visit at the CTC. Pills missed were determined by physical counting of the remained pills against the pills prescribed in the previous visit. Percentage (%) of pills missed was calculated by using the formulae below;

$$\%\;of\;pills\;missed=\frac{Number\;of\;pills\;remaining}{Total\;number\;of\;pills\;prescribed}\times 100$$

Percentage of drug adherence was calculated as follows;

%drugadherence=100−%ofpillsmissed

### Study population

The study population consisted of HIV-infected individuals attending the CTCs for routine care and who were on first-line ART treatment for one year or more. HIV-infected individuals with VF and viral suppression (VS) were included in the study as cases and controls respectively. The cases were defined as HIV-infected individuals with > 1000 copies/ml of HIV plasma RNA confirmed by < 0.5 logarithmic difference between initial and second HVL with three EAC in between. The controls were HIV-infected individuals attending the same CTCs along with cases but have viral suppression (HVL < 1000 copies/ml) that was measured in at least two different clinic visits. The cases found to have less than three EAC were excluded from the study. The study further excluded cases with a significant difference in HVL measured at the date of the interview and that measured before starting EAC (HIV-1 plasma RNA viral log_10_ drop greater than 0.5 at three-month interval with 3 EAC in between).

### Sample size determination

We assumed that the study had 80% power and the proportion exposed in the control group was 20%. With an equal number of cases and controls (ratio of 1:1), we could manage to detect an odds ratio (OR) of 3.0 or greater with the following sample size calculation as described by Charan and Biswas [13].

$$n=(\frac{r+1}{r})\frac{(\bar{p})(1-\bar{p}){\left(Z_{\beta }+Z_{\alpha /2}\right)}^{2}}{{\left(p_{1}-p_{2}\right)}^{2}}$$

*n* = sample size in each group, *r* = ratio of controls to cases, $\bar{p}$ = a measure of variability (average proportion exposed), *p*_**1**_ = proportion exposed in cases, *p*_2_ = proportion exposed in controls, *p*_1_ –*p*_2_ = Effect size (the difference in proportions), *Z*_*α/2*_ = level of two-tailed statistical significance, *Z*_*β*_ = standard normal variate for power of the study. Our study power was 80%, *Z*_*β*_ = 0.84; Level of significance was 0.05, *Z*_*α*_ = 1.96; *r* = 1 (equal number of cases and controls), *OR* = 3.0 and *p*_2_ = 20% or 0.2.

$$p_{1}=\frac{OR\times p_{2}}{p_{2}(OR-1)+1}=\frac{3.0\times 0.2}{0.2(3.0-1)+1}=0.43$$

$$\bar{p}=\frac{\left(p_{1}+p_{2}\right)}{2}=\frac{\left(0.43+0.2\right)}{2}=0.31$$

$$∴n=\frac{2\times (0.31)\times (1-0.31)\times {\left(0.84+1.96\right)}^{2}}{{\left(0.43-0.2\right)}^{2}}=63$$

The study thus aimed to recruit 63 cases and 63 controls making a total sample size of 126.

### Enrolment and study procedures

Cases were recruited consecutively after confirmation of VF in the CTCs. Systematic sampling was applied to recruit controls since nearly all of the CTC attendees at every clinic visit had viral suppression. Study participants who met the inclusion criteria and consented to participate were interviewed to collect demographic and clinical information. After the interviews, 8–10 ml of EDTA whole blood was collected and centrifuged at 2200 revolutions per minute for 10 minutes with brake-offs to separate plasma from buffy coat and red blood cells within 4 hours post-collection. HVL was enumerated in-vitro from plasma by reverse transcription-polymerase chain (PCR) reaction as per the Abbott m2000rt system and was expressed in copies/ml of plasma.

Due to financial constraints, 26 out of 63 samples of cases were selected for sequencing. The selected subset of samples represented the large cohort of 63 by age, duration on ART, gender, CD4 count, HAART in use, switching of ART, and HVL. The characteristics of the selected 26 samples and unselected 37 samples were similar (*p* > 0.05, Fisher’s Exact test) (S1 Table). Out of 63 cases, 37 (58.7%) were 35 or more years of age, of these, 16 (43.2%) were selected for sequencing. Out of 63 cases, 58 (92.1%) were on ART for more than 36 months, of these, 25 (43.1%) were selected for sequencing. Out of total 63 cases, 37 (58.7) were females, of these, 12 (32.4%) were chosen for sequencing. Thirty-three (53.2%) out of total 63 cases had CD4 count ≤ 350 cells/uL, of these, 16 (48.5%) were chosen for sequencing. The selected subset of samples had HVL > 14,000 copies/ml. S1 Table further describes the proportional characteristics of the selected and not selected samples for sequencing against the larger cohort of 63.

**Fig 1:**
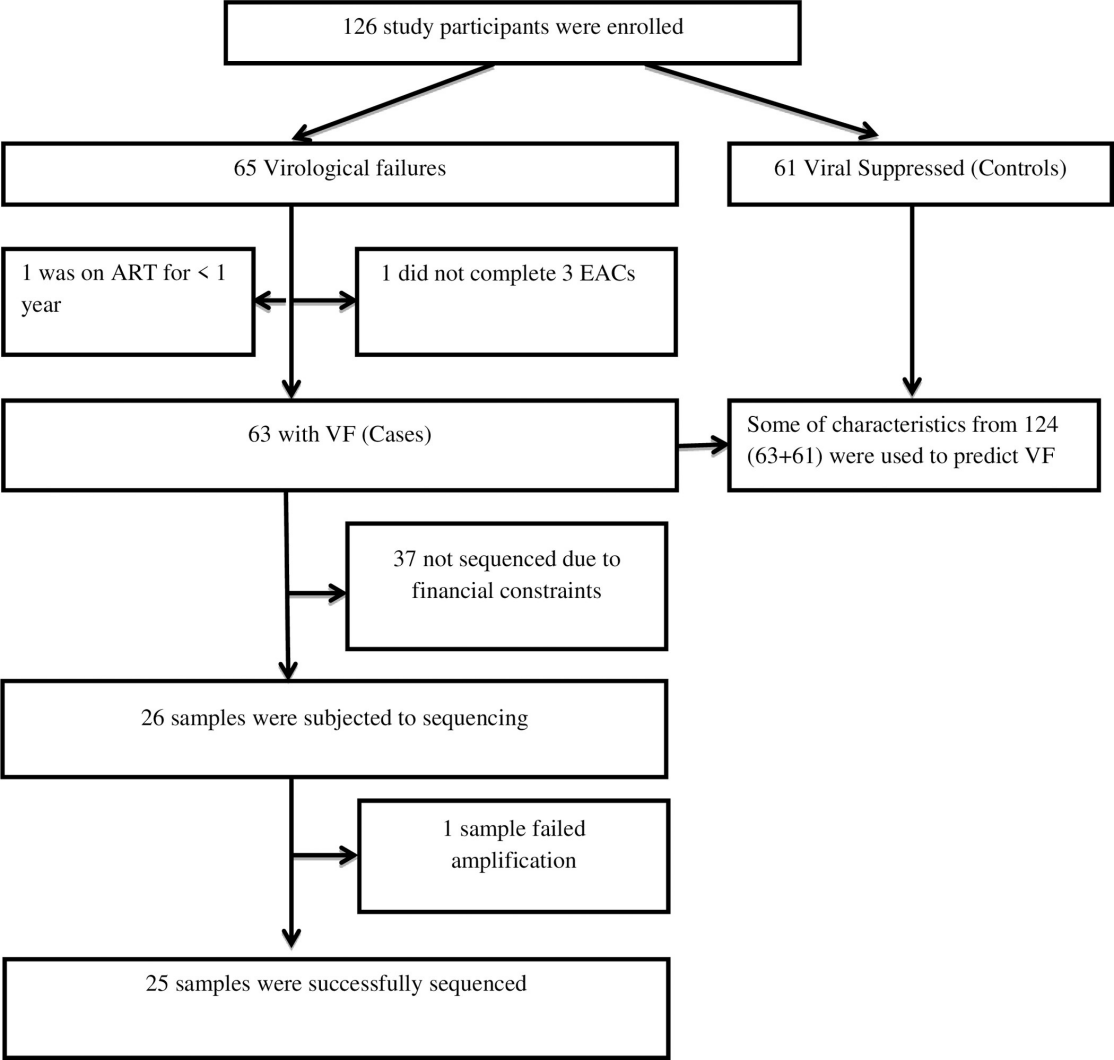
Study profile.

Plasma HIV-1 RNA was extracted using the PureLink® Viral RNA/DNA Mini Kit (Invitrogen, ThermoFisher Scientific, USA) as per manufacturer’s instructions. RT and protease genes of the extracted HIV-1 RNA were reversely transcribed into complementary DNA (cDNA) and nested PCR was done according to manufacturer’s instructions prescribed in the HIV-1 genotyping kit: Amplification module (Applied Biosystems, Life Technologies, Warrington, UK). The amplified cDNA was purified using the ExoSAP-IT™ PCR product clean-up reagent (Applied Biosystems, ThermoFisher Scientific, Inc.). Reactions were performed using the HIV-1 genotyping kit: Cycle sequencing module (Applied Biosystems, Life Technologies, Warrington, UK) based on Sanger sequencing method using BigDye™ Terminator v3.1 cycle sequencing kit (Applied Biosystems, ThermoFisher Scientific, Inc.). Sequencing was done using 3500xl genetic analyser (Applied Biosystems) with a 24 capillary 50 cm array. The study profile is described in **Fig 1**.

### Data analysis

#### Sequence data analysis

The raw sequence data were assembled, aligned and edited using automated base-calling software, RECall version 3.05 [14], available at (http://pssm.cfenet.ubc.ca) to generate individual consensus sequences. The sequence data were submitted to GenBank and obtained accession numbers MT347616–MT347640. HIVDRMs were analysed using the HIV drug resistance database of Stanford University available at (http://hivdb.stanford.edu) and HIVDRM mutation list from the 2019 updates of drug resistance (AIDS Society). To get HIV-1 subtypes, generated consensus sequences were analysed using REGA subtyping tool version 3.0 [15] available at (http://dbpartners.stanford.edu:8080/RegaSubtyping/stanford-hiv/typingtool/). REGA confirmed a subtype after clustering with a pure subtype in a database by > 800 base pairs with a bootstrap confidence of more than 70% in absence of recombination in the boot scan. The evidence for HIV-1 recombination was identified using Recombinant Identification Program version 3.0 [16] with window size 400 and 90% confidence threshold.

The maximum likelihood phylogenetic tree for HIV-1 diversity was generated using the Randomized Axelerated Maximum Likelihood (RAxML) program version 8.2.12 as previously described by Stamatakis [17]. Phylogenetic tree annotations were made using Interactive Tree Of Life (iTOL) [18] version 5 available at (https://itol.embl.de/).

#### Statistical analysis

Data analysis was performed using STATA version 14.0 (College Station, Texas 77845–4512, USA). Categorical variables were summarized using frequency and proportion while median with interquartile range was used to summarize numerical variables. A Chi-square test was used to compare the differences in proportions between groups. Odds ratios (OR) and 95% confidence interval (CI) for predictors of VF were estimated using multivariable logistic regression model. A *p*-value < 0.05 (2 tails) was considered statistically significant.

### Ethics consideration

The study was approved by the Kilimanjaro Christian Medical University College Research Ethics Review Committee in 2017 with ethical clearance certificate number 2028. The Kilimanjaro Regional Administrative Secretary granted permission to conduct the study at Pasua Health Centre, Majengo Health Centre and Mawenzi Regional Referral Hospital. The Executive Director of the KCMC granted permission to conduct the study at KCMC. All study participants consented/assented in writing to take part in the study. The HIV drug resistance testing results were shared with the respective CTCs for further management of the HIV-infected individuals.

## Results

### Demographic characteristics of study participants

A total of 124 study participants were recruited in this study. Out of the 124, 63 (50.8%) participants had VF and 61 (49.2%) had VS. The age of the 124 participants ranged from 15 to 79 years, with a median [IQR] age of 45 [35–52] years. Of the 124; 82 (66.1%) were females and 89 (71.8%) were employed in either formal or informal sector. The majority of the cases were 35 or more years of age and less educated (Table **1**).

**Table 1:** Demographic characteristics of study participants (N = 124).

	Overall	VF (Cases)	VS (Controls)	*p-*value^b^
Characteristic	n (%)	n (%)	n (%)	
Age (years)				
15–34	29 (23.4)	26 (41.3)	3 (4.9)	< 0.001
≥ 35	95 (76.6)	37 (58.7)	58 (95.1)	
Median age [IQR] *^a^*	45 [35–52]	41 [21–49]	48 [43–54]	
Sex				
Male	42 (33.9)	26 (41.3)	16 (26.2)	0.077
Female	82 (66.1)	37 (58.7)	45 (73.8)	
Education level				
Primary or none	84 (67.7)	36 (57.2)	48 (78.7)	0.010
Secondary and above	40 (32.3)	27 (42.8)	13 (21.3)	
Marital status				
Married/cohabiting	40 (32.3)	18 (28.6)	22 (36.1)	0.283
Single	84 (67.7)	45 (71.4)	39 (63.9)	
Occupation				
Employed	89 (71.8)	36 (57.1)	53 (86.9)	< 0.001
Unemployed	35 (28.2)	27 (42.9)	8 (13.1)	
CTC enrolled				
KCMC	53 (42.7)	38 (60.3)	15 (24.6)	< 0.001
Majengo HC	19 (15.3)	7 (11.1)	12 (19.7)	
Mawenzi RRH	29 (23.4)	14 (22.2)	15 (24.6)	
Pasua HC	23 (18.6)	4 (6.4)	19 (31.1)	

CTC = Care and treatment clinic, KCMC = Kilimanjaro Christian Medical Centre, HC = Health Centre, RRH = Regional Referral Hospital, IQR = Interquartile Range, VF = Virological failure, VS = Viral suppression;

a = Expressed as median [Interquartile range],

b = χ^2^ test.

### Clinical characteristics of study participants

The median [IQR] length of time on ART for the 124 participants was 72 [48–104] months, 92 (74.2%) had good adherence, 65 (52.4%) were in non-tenofovir based HAART and 66 (53.2%) were in WHO clinical stage III/IV (Table **2**). The median [IQR] CD4 count (cells/μL) at follow up was higher in VS participants 518 [326–741] than in VF participants 334 [134–549]. Likewise, 100% of VS participants reported good adherence to ART compared to 49% of VF participants (Table **2**).

**Table 2:** Clinical characteristics of study participants (N = 124).

Characteristic	Overall n (%)	VF (Cases) n (%)	VS (Controls) n (%)	*p-*value^b^
Duration on ART (months)				
12–36	20 (16.1)	7 (11.1)	13 (21.3)	0.094
> 36	104 (83.9)	56 (88.9)	48 (78.7)	
Median [IQR]	72 [48–104]	84 [60–120]	72 [48–96]	
Adherence status				
Good	92 (74.2)	31 (49.2)	61 (100.0)	< 0.001
Poor	32 (25.8)	32 (50.8)	0 (0.0)	
Ever switched ART				
Yes	54 (43.5)	30 (47.6)	24 (39.3)	0.353
No	70 (56.5)	33 (52.4)	37 (60.7)	
HAART initiated				
TDF based	39 (31.5)	12 (19.0)	27 (44.3)	0.009
Non-TDF based	85 (68.5)	51 (81.0)	34 (55.7)	
HAART in use				
TDF based	59 (47.6)	22 (34.9)	37 (60.7)	0.012
Non-TDF based	65 (52.4)	41 (65.1)	24 (39.3)	
Baseline CD4 count (cells/μL)				
< 100	37 (29.8)	17 (27)	20 (32.8)	0.480
≥ 100	87 (70.2)	46 (73)	41 (67.2)	
Median [IQR]	173 [72–276]	179 [68–256]	169 [73–282]	
Follow up CD4 count (cells/μL)				
< 100	13 (10.5)	12 (19)	1 (1.6)	0.002
≥ 100	111 (89.5)	51(81)	60 (98.4)	
Median [IQR]	444[215–628]	334 [134–549]	518 [326–741]	
WHO clinical stage				
I/II	8 (46.8)	26 (41.3)	32 (52.5)	0.212
III/IV	66 (53.2)	37 (58.7)	29 (47.5)	
>Time from HIV diagnosis to ART initiation (months)^a^		3.5 [1.4–17.9]	2.6 [0.9–14.0]	

TDF = Tenofovir Disoproxil Fumarate, ART = Antiretroviral therapy, HAART = Highly Active Antiretroviral Therapy, IQR = Interquartile Range, WHO = World Health Organisation, VF = Virological failure, VS = Viral suppression,

a = Expressed as median [Interquartile range],

b = χ^2^ test.

### Characteristics of sequenced samples

**Fig 2:**
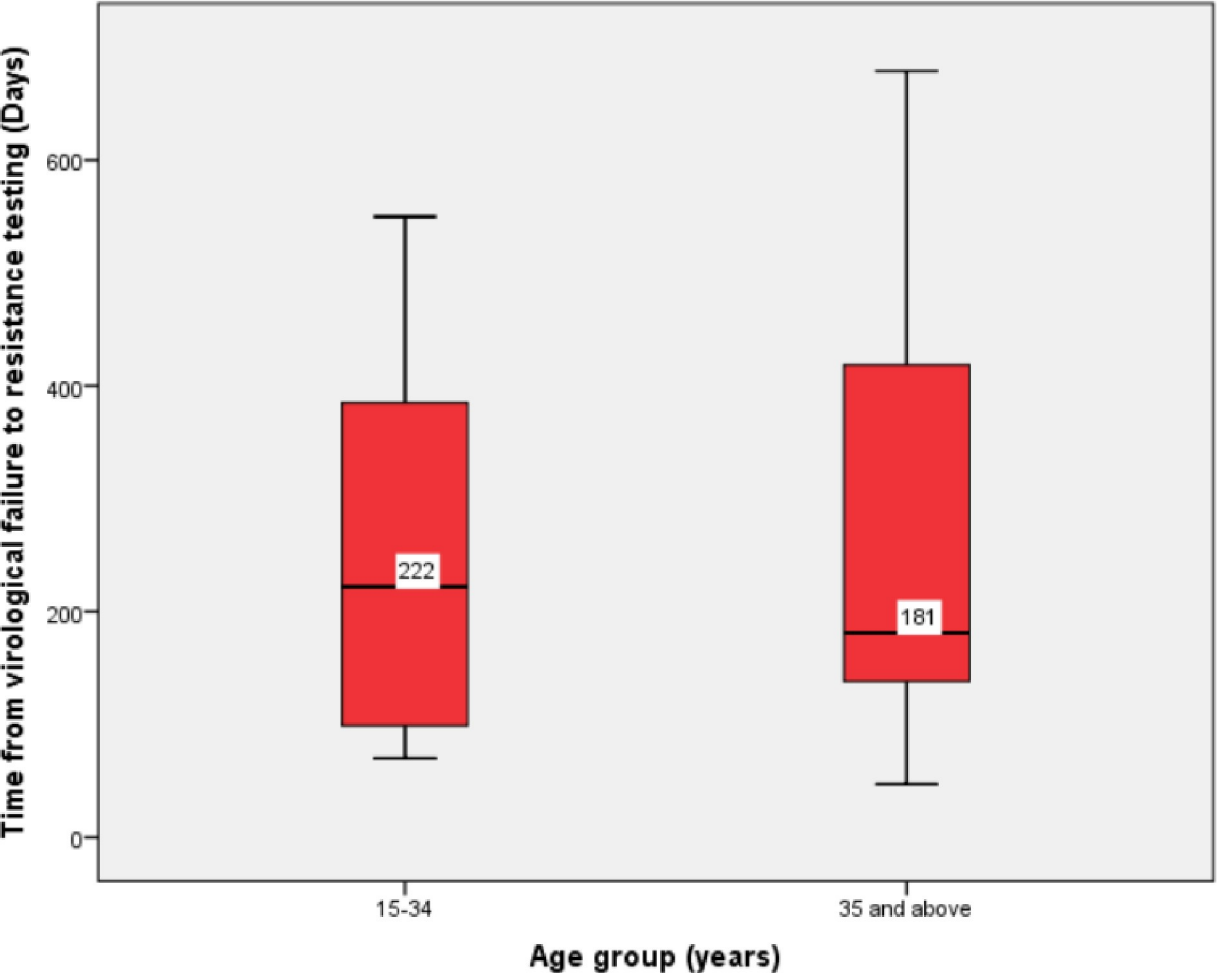
Time from virological failure to HIV drug resistance testing.

Among cases whose samples were sequenced, the median [IQR] length of time from VF to HIV drug resistance testing was 190 [115–392] days. The median [IQR] length of time from VF to HIV drug resistance testing was 222 [94–387] days among the participants aged between 15 and 34 years compared to their counterparts with 181 [132–432] days (Fig 2). Sequencing of RT and protease genes of HIV-1 was successful on 25 out of 26 selected samples, one sample failed amplification. Of the 25 samples, almost all (n = 24) had at least one major mutation conferring resistance to HIV drug (Table 3). Dual-class resistance was observed in 16 (64%) samples. In general, thirteen samples (52%) had at least one thymidine analogue- resistance associated mutation (TAM) while three samples (12%) had T69D mutation together with at least 1 TAM. Identified TAMs were T215YF (n = 9), K219QE (n = 7), K70R (n = 7), D67N (n = 6), M41L (n = 4), and L210W (n = 3) (Table 3). The presence of T69D mutation along with at least 1 TAM reduces the susceptibility of all currently NRTIs in use. One sample was fully susceptible (no detectable HIV drug resistance mutation) despite having VF and substantial-high HVL at the date of the interview.

**Table 3:** Characteristics of successfully sequenced samples (N = 25).

SN	Sample ID	GenBank accession numbers	Age (years)	Duration on ART (years)	HAART at date of interview	HAART previously consumed	HVL at date of interview	NRTI resistance associated mutations	NNRTI resistance associated mutations	PI resistance mutations	HIV-1 subtype
1	K03	MT347616	18	10	AZT+3TC+EFV	d4T+3TC+EFV	389,006	M184V, D67T, K70R,T215AITV, K219E	Y188L, K238T	None	A1
2	K11	MT347617	55	6	ABC+3TC+LPVr	AZT+3TC+EFV	24,463	D67N, K70R, M184V,K219E, T215F	None	L10F,K20T, M46I,I47V,N88G	C
3	K12	MT347618	29	6	AZT+3TC+EFV	AZT+3TC+EFV	20,805	M184V	K103N,V108I, K238T	None	CRF10_CD
4	K15	MT347619	49	12	AZT+3TC+NVP	d4T+3TC+NVP	81,123	None	None	None	A1
5	K16	MT347620	44	3	TDF+3TC+EFV	None	23,354	None	K103N	None	A1
6	K17	MT347621	15	7	AZT+3TC+NVP	None	25,879	None	G190A	None	A1
7	K19	MT347622	17	6	ABC+3TC+ LPVr	ABC+3TC+EFV	296,646	M41L, D67N, K70R, M184V, T215Y, K219Q, L74I,T69D	A98G,K103N, P225H,F227F, 238T	None	C
8	K21	MT347623	20	13	ZVD+3TC+EFV	None	33,887	M184V,T215F	K103N,Y188F, M230L	None	C
9	K25	MT347624	19	10	AZT+3TC+EFV	None	37,794	None	V106M, G190A	None	C
10	K29	MT347625	34	9	TDF+FTC+ATVr	AZT+3TC+EFV	14,431	None	K103S, V106M	None	C
11	K32	MT347626	41	4	TDF+3TC+EFV	None	161,220	A62V, K65R, M184V,K219E	K101E,V106M, Y181C, G190A	None	C
12	K36	MT347627	29	10	AZT+3TC+EFV	None	23,056	M184V	L100I, K103N	None	A1
13	K37	MT347628	15	9	AZT+3TC+NVP	d4T+3TC+NVP	20,540	D67N, K70R, M184V,T215F, K219Q	A98G, K101E, G190A	None	C
14	K38	MT347629	46	13	ABC+3TC+ATVr	TDF+FTC+LPVr	1,635,827	M41L, E44D, D67N, T69D, M184V,L210W, T215Y, K219R	K101E, E138K, Y181C, G190A, H221Y	L24I, L33F, M46I, I54V, Q58E,V82A, N88S	CRF35_AD
15	M02	MT347630	35	5	AZT+3TC+NVP	None	124,714	D67N, T69D, K70R, M184V	G190A	None	D
16	M03	MT347631	69	9	AZT+3TC+NVP	None	36,600	M41L, E44A, M184V,L210W, T215Y, K219N	Y181C, H221Y	None	A1
17	M05	MT347632	53	10	AZT+3TC+NVP	d4T+3TC+NVP	65,638	D67G, K70R, M184V,T215FV, K219E	K103S, E138Q	None	A1
18	M07	MT347633	52	4	ABC+3TC+EFV	None	66,486	M184V	K103N	None	A1
19	M10	MT347634	41	8	AZT+3TC+NVP	None	45,819	D67N, K70R, M184V,K219Q	K103N, V179L, K238T	None	A1
20	MJ01	MT347635	48	9	AZT+3TC+EFV	None	14,065	M184V,L210W, T215Y	K103N	None	C
21	MJ03	MT347636	36	9	TDF+3TC+EFV	None	176,378	None	K103N	None	C
22	MJ05	MT347637	45	5	TDF+FTC+EFV	None	222,227	None	L100I, K103N	None	C
23	MJ06	MT347638	51	4	TDF+3TC+EFV	AZT+3TC+NVP	14,711	K70E, M184V	K103N,V108I, H221Y, F227L	None	D
24	P02	MT347639	54	8	AZT+3TC+EFV	None	235,443	None	V106M, V179D	None	C
25	P03	MT347640	45	5	TDF+3TC+EFV	AZT+3TC+NVP	128,781	M41L, K70S, L74I, M184V, T215Y	V106M,E138Q, F227L	None	C

### Profiles of acquired HIV drug resistance in RT and protease genes

**Fig 3:**
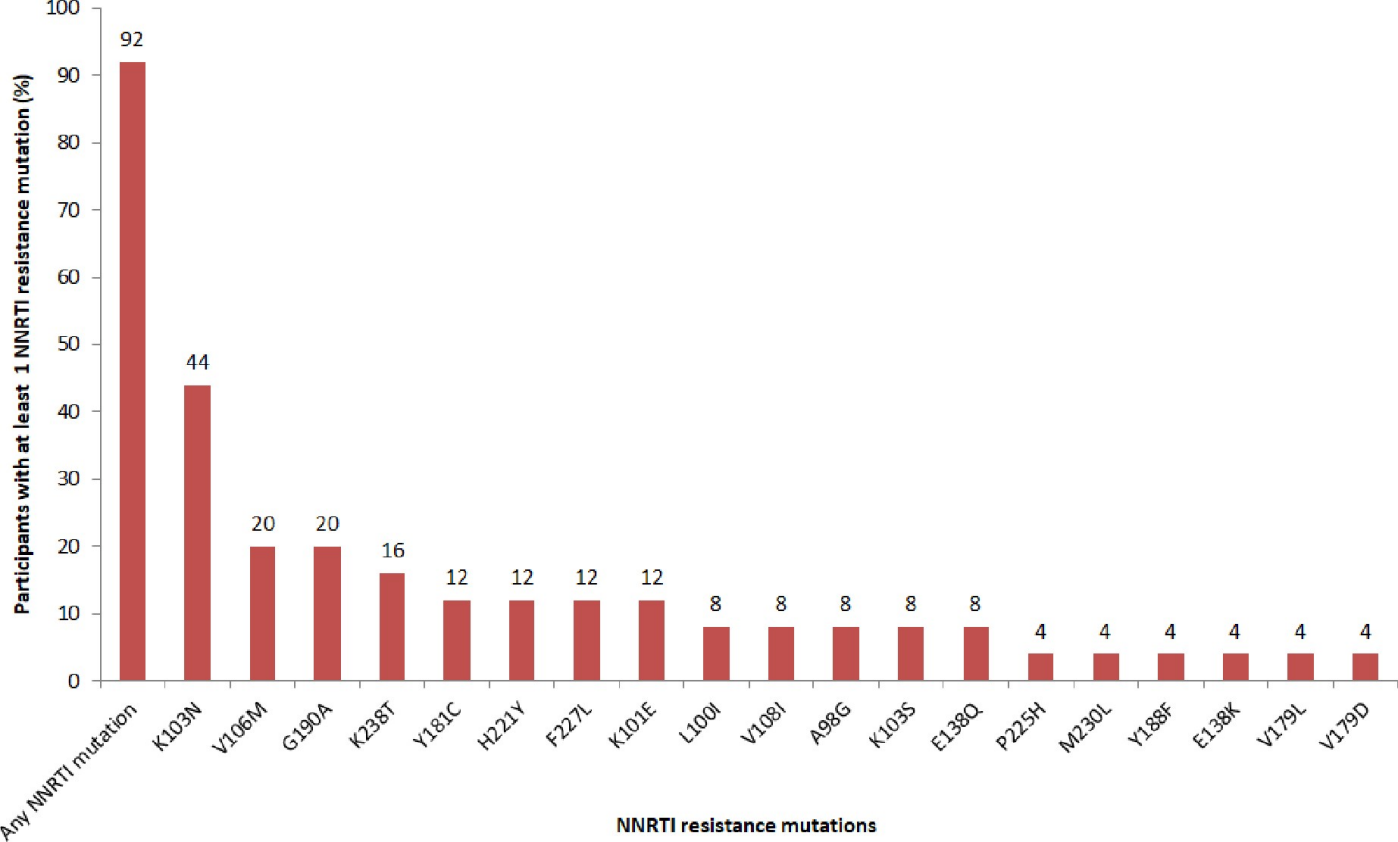
Profiles of NNRTI resistance mutations in participants with virological failure.

Out of 25 successfully sequenced samples, 23 (92%) samples had at least one NNRTI resistance-associated mutations (**Fig 3**). The most frequent NNRTI resistance-associated mutations were K103N (44%), V106M (20%), and G190A (20%) (**Fig 3**), all of these three mutations confer high-level resistance to efavirenz and nevirapine.

**Fig 4:**
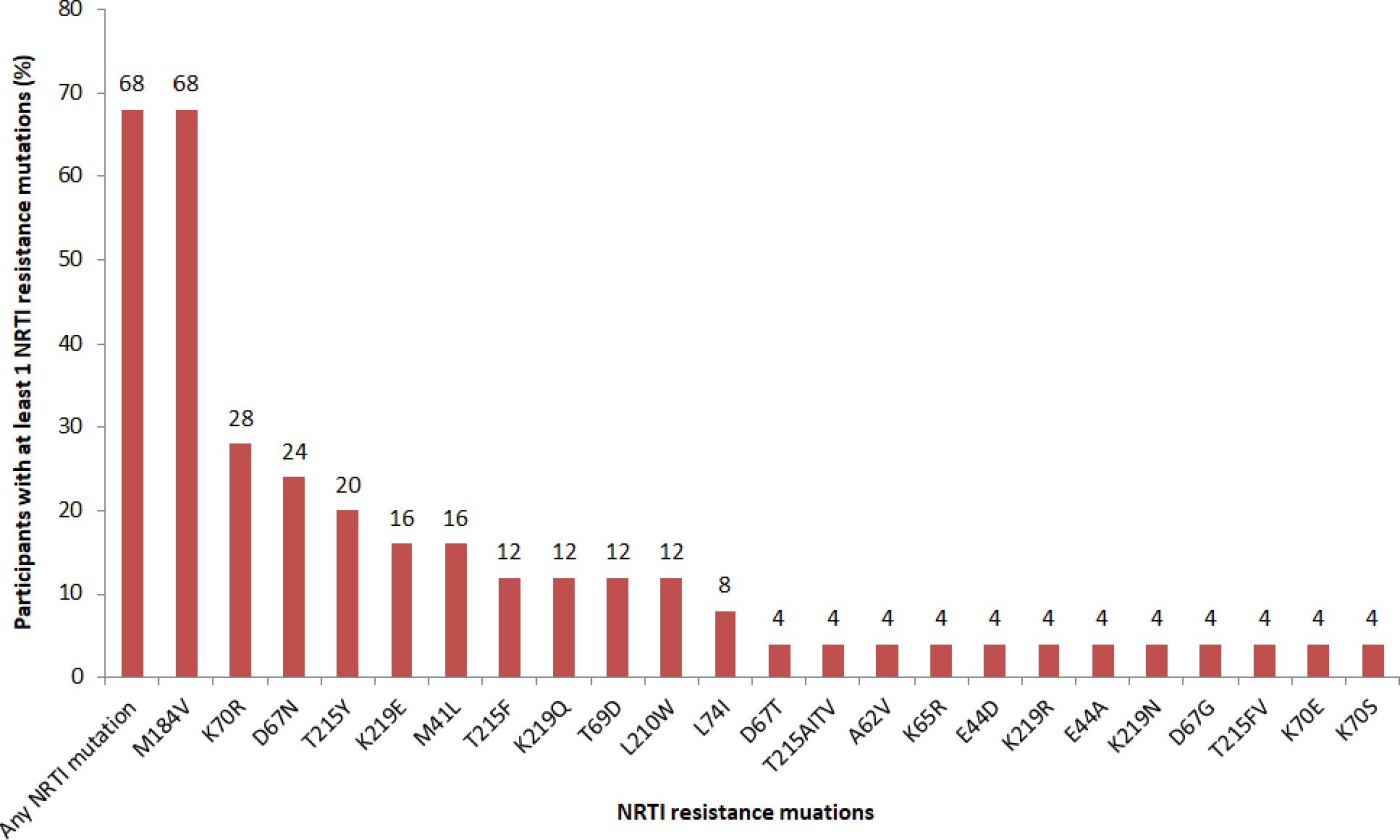
Profiles of NRTI resistance mutations in participants with virological failure.

Seventeen samples (68%) had at least one mutation associated with NRTI resistance. The most frequent NRTI resistance-associated mutations were M184V (68%), K70R (28%), and D67N (24%) (**Fig 4**). M184V mutation was associated with high-level resistance to emtricitabine, and lamivudine; while K70R and D67N are among thymidine analogue-associated resistance mutations (TAM) reducing the susceptibility of all current NRTI in use.

Two samples (8%) had at least one mutation associated with protease inhibitors (PI) resistance (Table 3). One participant (K38) who had previously used ritonavir-boosted lopinavir had L24I, L33F, I54V, and V82A mutations which together reduce the susceptibility of ritonavir-boosted lopinavir. The same participant (K38) who was on ritonavir-boosted atazanavir at the date of interview had N88S mutation which confers a great effect on atazanavir. Only M46I mutation was found in both samples.

### HIV-1 diversity

**Fig 5:**
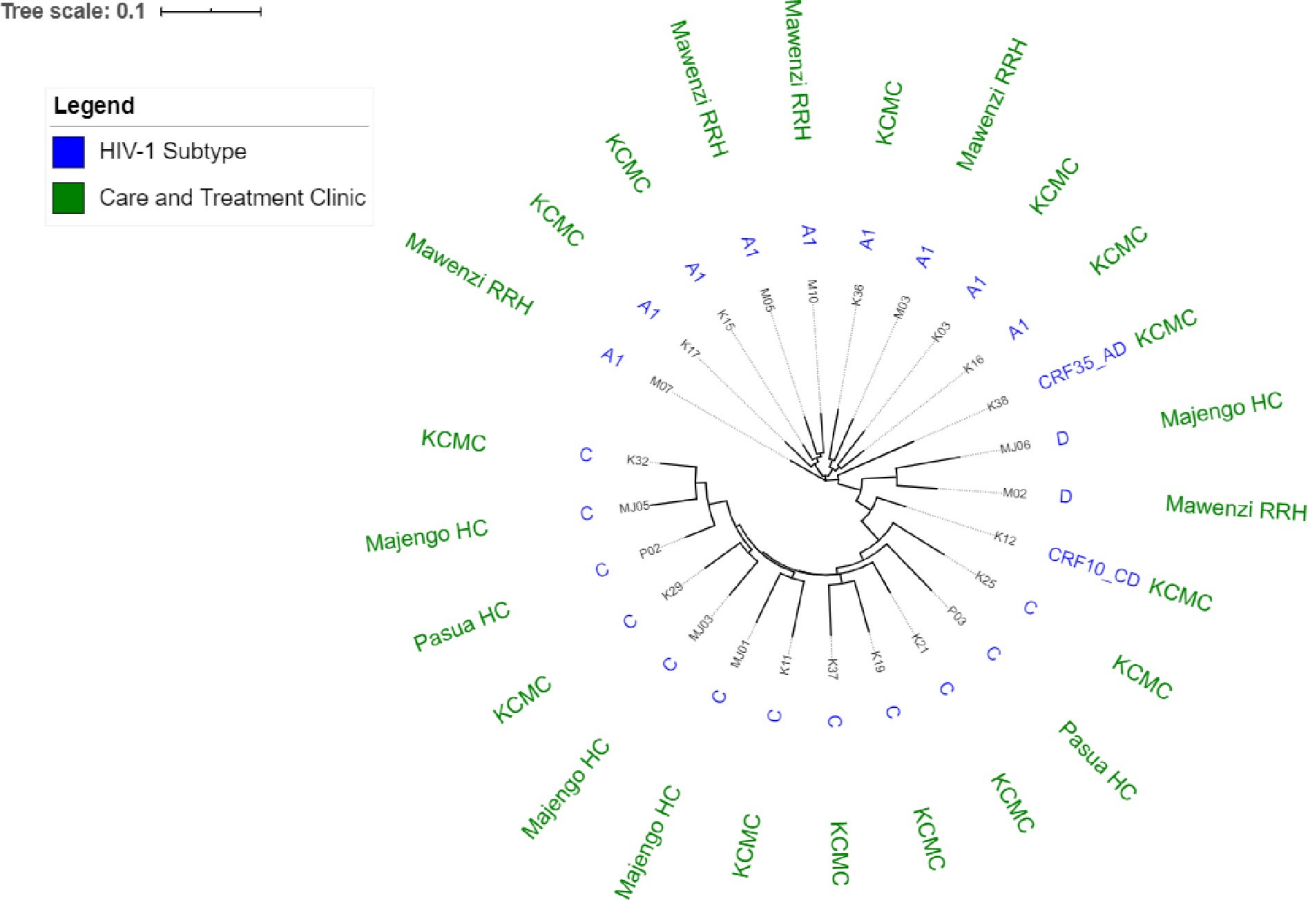
A maximum-likelihood phylogenetic tree.

The proportions of HIV-1 diversity in the RT and protease genes are shown in Table 4. HIV-1 subtype C was the most prevalent subtype (48%) followed by A1 (36%), D (8%) and Circulating Recombinant Forms (CRF) (8%). The recombinants identified were CRF10_CD and CRF35_AD. A maximum-likelihood phylogenetic tree (Fig 5) describes the HIV-1 diversity with annotations. The two CRFs were only reported at KCMC. Subtype C was reported in all study sites except Mawenzi RRH (Fig 5).

**Table 4:** HIV-1 diversity (N = 25).

Subtype	Number of sequences	Percentage
C	12	48
A (A1)	9	36
D	2	8
CRF35_AD	1	4
CRF10_CD	1	4
**Total**	**25**	**100**

### Predictors of VF

The predictors of VF are shown in Table **5**. In bivariate analysis, participant age, occupation, HAART initiated and HAART in use were significantly associated with VF. In the multivariable logistic regression analysis, occupation had marginal statistical significance [aOR = 0.35, 95% CI (0.12–1.04), *p* = 0.059] while age was an independent predictor of VF. Being employed in either formal or informal sector was 65% protective against VF. Furthermore, one unit increase in participant age (year) was associated with 6% lower odds of VF [aOR = 0.94, 95% CI (0.90–0.97), *p* < 0.001].

**Table 5:** Predictors of VF (N = 124).

Characteristic	VF		Crude analysis		Multivariable analysis	
	No (%)	Yes (%)	cOR (95% CI)	*p-*value	aOR (95% CI)	*p-*value
Age [years]*	48 [43–54]	41 [21–49]	0.93 (0.90–0.96)	<0.001	0.94 (0.90–0.97)	0.001
Sex						
Male	16 (26.2)	26 (41.3)	1		1	
Female	45 (73.8)	37 (58.7)	0.51 (0.24–1.08)	0.079	0.56 (0.21–1.51)	0.254
Marital status						
Married	22 (36.1)	18 (28.6)	0.71 (0.33–1.51)	0.373	-	-
Single	39(63.9)	45 (71.4)	1			
Occupation						
Employed	53 (86.9)	36 (57.1)	0.2 (0.08–0.49)	<0.001	0.35 (0.12–1.04)	0.059
Unemployed	8 (13.1)	27 (42.9)	1		1	
Duration on ART (months)						
12–36	13 (21.3)	7 (11.1)	1			
> 36	48 (78.7)	56 (88.9)	2.17 (0.80–5.87)	0.128	-	-
Switched ART						
Yes	24 (39.3)	30 (47.6)	1.4 (0.69–2.86)	0.353	0.89 (0.31–2.53)	0.827
No	37 (60.7)	33 (52.4)	1		1	
HAART initiated						
TDF based	27 (44.3)	12 (19)	0.3 (0.13–0.66)	0.003	0.26 (0.05–1.27)	0.096
Non-TDF	34 (55.7)	51 (81)	1		1	
HAART in use						
TDF based	37 (60.7)	22 (34.9)	0.35 (0.17–0.72)	0.005	0.85 (0.25–2.85)	0.795
Non-TDF	24 (39.3)	41 (65.1)	1		1	
Baseline CD4 count (cells/μL)						
< 100	20 (32.8)	17 (27)	0.76 (0.35–1.64)	0.481	0.86 (0.35–2.11)	0.745
≥ 100	41 (67.2)	46 (73)	1		1	
WHO clinical stage at interview						
I/II	32 (52.5)	26 (41.3)	1		1	
III/IV	29 (47.5)	37 (58.7)	1.57 (0.77–3.19)	0.213	1.04 (0.44–2.46)	0.931

* Median age [IQR], cOR: Crude odds ratio, aOR: Adjusted odds ratio.

Despite being statistically not significant, participants with VF had 1.04 (0.44–2.46) odds of being at WHO clinical stage III/IV. This finding concurs with the well-known positive association between VF and an increase in WHO clinical stage.

## Discussion

This study aimed at determining HIVDRMs and predictors of VF in HIV-infected individuals failing to respond to first-line ART in Moshi municipality, Northern Tanzania. High profiles of HIVDRMs conferring acquired HIV drug resistance to NRTIs, NNRTIs and PIs were found. While occupation had marginal statistical significance in predicting VF, age was an independent factor associated with VF.

Most (96%) of the successfully sequenced samples had at least one major mutation conferring resistance to HIV drugs. NNRTI resistance (92%) was greater than NRTIs resistance (68%) consistent with findings elsewhere [6]. The most frequent NNRTI resistance-associated mutations were K103N, V106M, and G190A that confer high-level resistance to efavirenz and nevirapine [19]. NNRTIs were previously reported to have a low genetic barrier and hence highly vulnerable to resistance [20]. These findings further support the Tanzanian programmatic intervention to replace efavirenz with dolutegravir which has proven to have a high genetic barrier to resistance [21]. This study further reports frequent NRTI resistance-associated mutations as M184V, K70R, and D67N in line with other studies [10]. M184V associates with high-level resistance to abacavir, emtricitabine, and lamivudine; while K70R and D67N are among the TAMs. More than a half of sequenced samples had at least one TAM. Existence of TAMs reduces the susceptibility of all available NRTIs with an exception of emtricitabine and lamivudine [22].

This study reports co-existence of T69D mutation with at least one TAM in three samples. Two of these samples had M41L and T215Y TAMs, a combination which further enhances cross-resistance to all US FDA approved NRTIs, a phenomenon known as Multi-NRTI resistance [19, 23]. As a result, the Tanzania National AIDS Control Program may have fewer options of main-stream HIV drugs both in first and second-line regimens. Continuous programmatic monitoring of HIV drug resistance in HIV-infected individuals failing to respond to first-line HIV drugs will preserve the integrity of few NRTI options in second-line. However, there is a need to scale-up NRTIs with substantial genetic barrier to resistance.

This study describes full genotypic susceptibility of prescribed HAART to a 49 years old individual with ID K15, on ART for 12 years and with HVL 81,123 copies/ml. At the interview, he reported good adherence to the prescribed HAART. Although the participant self-reported good adherence to medication, we speculate the reality of adherence was low. Several factors hinder adherence to medications including stigma, and lack of social support [24]. Community-based HIV treatment supporters chosen by HIV-infected individuals may improve adherence to medications and lead to favourable treatment outcomes [25].

A significant association between age and VF concurs with findings from numerous countries in Western, Southern and Eastern Africa [26–33]. HIV-infected adolescents and young adults on HAART previously reported to have inconsistency in adhering to antiretroviral medication due to anxiety, depression, forgetfulness, fear of disclosure, ART adverse events and abandoning medication when they feel better [34], consequences which contribute to VF. Therefore, special care and treatment for adolescents and young adults are paramount with an emphasis on health education particularly the importance of disclosure and adhering to medications.

This study reports that HIV drug-experienced individuals in formal or informal employment were protected from VF. Unemployment among HIV-infected individuals was previously described as a stressful event which reduced adherence to prescribed HIV medications resulting in high rates of VF [35] and increased mortality [36]. Supporting and engaging PLHIV in income-generating activities may stabilize financial and social status and positively impact drug adherence and favourable treatment outcomes.

Although not statistically significant, this study reports the positive association between advancement of WHO clinical stage of AIDS and VF, a finding which was consistent with those reported by Jobanputra et al in (2015) [28]. VF in individuals with advanced HIV/AIDS can be explained by the severe state of immunodeficiency which attracts opportunistic infections (OIs) and replicative fitness of HIV [37]. Early diagnosis of HIV, effective treatment and monitoring of HIV/AIDS and related OIs among individuals on HAART is highly recommended [9].

Tenofovir (TDF)-based combination antiretroviral therapy is one of the recommended first-line antiretroviral drugs of choice for adolescents and adults living with HIV worldwide [9] as well as in Tanzania [8]. Although not statistically significant, being on TDF-based first-line HAART was protective against VF, this is consistent with studies previously done in North America [38], Thailand [39] and in a systematic review [40]. TDF-based regimens were extensively reported in previous randomized clinical trials to be more effective than other combination therapies in bringing favourable virological and immunologic outcomes [41, 42]. A possible explanation is that, compared to other back-borne antiretroviral drugs, TDF-based therapy is consumed once daily with minimal treatment-related toxicity [41, 43], is more tolerable [44] and can be easily adhered to by PLHIV [45]. Therefore we complement the prescription of TDF-based HAART in PLHIV initiating HAART as the strategy of reducing VF.

### Limitations of the study

One of the limitations of this study is reporting bias of adherence to HAART and pill counting at the clinic visit. Some of the study participants might have provided imprecise information about adherence. However, this was the common practice prescribed under the Tanzanian HIV and AIDS Treatment and Management Guidelines of October 2017. Also, recall biases are certain to some of the information requested from the VS group.

Finally, the study would have had much more to offer by sequencing the entire cohort of 63 plasma samples from participants with VF. Unfortunately, due to financial constraints, only 26 out of 63 plasma samples were sequenced. However, selection of a subset of 26 samples proportionally represented the large cohort 63 by age, duration on ART, gender, CD4 count, HAART in use, switching of ART, and HVL.

## Conclusion

There is a high rate of HIVDRMs in HIV-infected individuals failing to respond to first-line HAART in Moshi, Northern Tanzania. This is probably a result of no programmatic monitoring of HIV drug resistance in individuals with VF. Prompt intervention is required to safeguard the limited number of second-line HIV drug options, such as the implementation of HIV drug resistance monitoring before and after switching HAART. In addition, scale-up of HIV drugs with higher genetic barrier to resistance is of importance.

## Supporting information

S1 Dataset Demographic and clinical data. (XLSX)

S1 Table Characteristics of sequenced and not sequenced samples against total samples from virological failures. (DOCX)
