# Supplementary material for: Patterns of acquired HIV-1 drug resistance mutations and predictors of virological failure in Moshi, Northern Tanzania
Source: PLoS One. 2020 Sep 28;15(9):e0232649. doi: 10.1371/journal.pone.0232649 (PMC7521739; doi:10.1371/journal.pone.0232649)
Supplement: S1 Table — (DOCX) [file pone.0232649.s002.docx]

| S1 Table. Characteristics of Sequenced and not sequenced samples against total samples from virological failures (Cases) (N=63) | | | | |
| --- | --- | --- | --- | --- |
| **Characteristic** | **Total cases** | **Sequenced** | **Not sequenced** | ****p*-value** |
|  | **n (%)** | **n (%)** | **n (%)** |  |
| Age (years) |  |  |  |  |
| 15-34 | 26 (41.3) | 10 (38.5) | 16 (43.2) | 0.797 |
| ≥35 | 37 (58.7) | 16 (61.5) | 21 (56.8) |  |
| Sex |  |  |  |  |
| Male | 26 (41.3) | 14 (53.8) | 12 (32.4) | 0.121 |
| Female | 37 (58.7) | 12 (46.2) | 25 (67.6) |  |
| Duration of ART intake (months) |  |  |  |  |
| 12 – 36 | 5 (7.9) | 1 (3.8) | 4 (10.8) | **0.394 |
| >36 | 58 (92.1) | 25 (96.2) | 33 (89.2) |  |
| HAART on use |  |  |  |  |
| TDF based | 21 (33.3) | 6 (23.1) | 15 (40.5) | 0.182 |
| Non TDF based | 42 (66.7) | 20 (76.9) | 22 (59.5) |  |
| Ever Switched ART |  |  |  |  |
| Yes | 30 (47.6) | 10 (54.1) | 20 (38.5) | 0.306 |
| No | 33 (52.4) | 16 (61.5) | 17 (45.9) |  |
| CD4 Count |  |  |  |  |
| ≤350 | 33 (53.2) | 16 (61.5) | 17 (47.2) | 0.310 |
| >350 | 29 (46.8) | 10 (38.5) | 19 (52.8) |  |
| HAART=Highly Active Antiretroviral Therapy, ART=Antiretroviral Therapy, TDF=Tenofovir Disoproxil Fumarate  * Fisher’s Exact Test  ** Two cells had expected counts less than 5 | | | | |
